# Non–component clinical feature–based machine learning for disease activity risk stratification in juvenile idiopathic arthritis: a multi–center retrospective validation study

**DOI:** 10.3389/fped.2026.1820800

**Published:** 2026-07-15

**Authors:** Peipei Dong, Fei Song, Bin Wang, Song Gao, Hongyang Dong, Xiaohong Jiang, Yan Cong, Chuansheng Wu

**Affiliations:** 1 Pediatric Department, Lianyungang Hospital of Traditional Chinese Medicine, Lianyungang, Jiangsu, China; 2 Traditional Chinese Medicine Department, The First People’s Hospital of Lianyungang, Lianyungang, Jiangsu, China; 3 Department of Rheumatology, Lianyungang Hospital of Traditional Chinese Medicine, Lianyungang, Jiangsu, China

**Keywords:** disease activity prediction, JADAS27, juvenile idiopathic arthritis, multi–center study, non–component features, risk stratification

## Abstract

**Background:**

JADAS27 is widely used to assess juvenile idiopathic arthritis (JIA) disease activity, but complete scoring is impractical in many clinical settings because it requires simultaneous physician global assessment (PhGA), patient/parent global assessment (PtGA), active joint count (AJC), and erythrocyte sedimentation rate (ESR). We asked whether non–component clinical variables alone–strictly excluding all four JADAS27 constituents–can classify JADAS27–defined activity strata.

**Methods:**

In this retrospective multi–center study, 800 patients with JIA were enrolled from CARRA (*n* = 400), PRCSG (*n* = 240), and LHTCM (*n* = 160). Disease activity was categorized as inactive, low, moderate, or high by established JADAS27 cutoffs. PhGA, PtGA, AJC, and ESR were excluded from all model inputs to prevent circular prediction. Thirteen remaining non–component variables (18 encoded features) were used to train four machine learning algorithms on CARRA + PRCSG (*n* = 640), with independent external validation on LHTCM (*n* = 160). Pre–specified ablation analyses quantified contributions of proxy variables (CHAQ, pain score, limited joint count).

**Results:**

SVM achieved the best external validation performance (accuracy 0.731, 95% CI 0.657–0.797; macro AUC 0.918, 95% CI 0.887–0.945). Class–wise recall was highest for inactive (82.4%) and high activity (77.4%), with most errors between adjacent classes. SHAP and permutation importance analyses consistently identified CHAQ score, JIA subtype, pain score, limited joint count, and C–reactive protein as the most influential predictors. Removing all three proxy variables reduced accuracy to 0.619 and macro AUC to 0.843, indicating that both proxy and non–proxy features contribute independently. Sensitivity analysis with subtype–specific cutoffs yielded comparable performance (accuracy 0.713).

**Conclusion:**

Strictly non–component clinical features can stratify JADAS27–defined disease activity with clinically meaningful external performance. This approach may support early risk stratification when formal JADAS27 scoring is unavailable, and complements rather than replaces physician assessment.

## 1 Introduction

Juvenile idiopathic arthritis (JIA) is the most common chronic pediatric rheumatic disease and affects roughly 1 in 1,000 children worldwide (1, 2). Because JIA includes seven ILAR subtypes with distinct trajectories and severity, treatment requires frequent and reliable disease activity assessment (3, 4). In routine practice, JADAS27 is a key composite metric integrating physician global assessment, patient/parent global assessment, active joint count, and ESR (5, 6). However, complete JADAS27 scoring is not always feasible, particularly in resource–limited clinics, urgent visits, and telemedicine.

Machine learning (ML) can model nonlinear, high–dimensional clinical relationships and has shown value in adult rheumatology (7–9). In pediatric rheumatology, evidence remains limited, especially for multiclass JIA activity classification. A key methodological risk is circular prediction: including JADAS27 components as predictors for a JADAS27–defined target would largely reconstitute the formula rather than provide clinically useful inference.

To address this issue, we developed models using only non–component variables. We also recognized that some non–component features (CHAQ, pain score, limited joint count) are correlated with JADAS27 components and may act as partial proxies. Therefore, we pre–specified ablation analyses to quantify how much predictive performance derives from these proxy variables vs. independent clinical indicators.

**Scheme 1:**
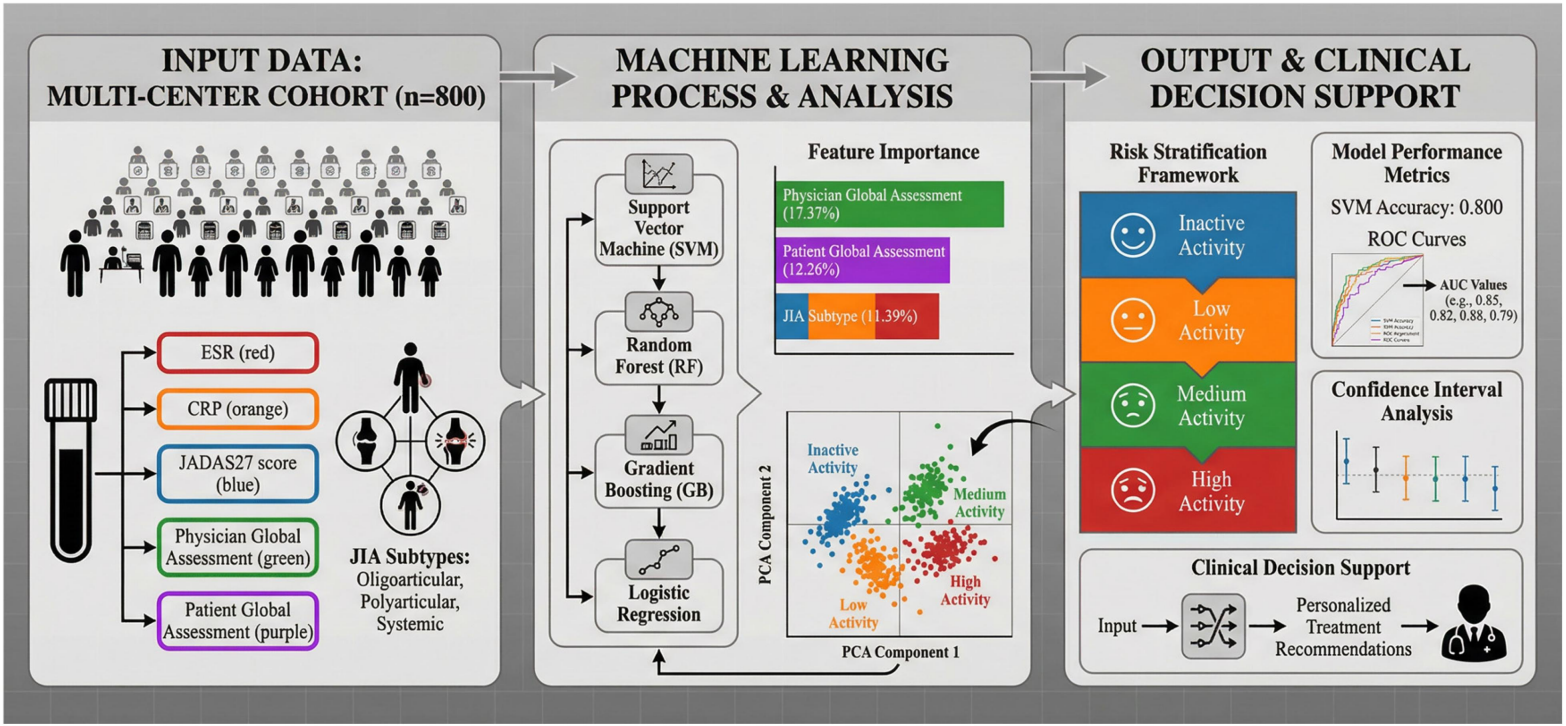
Schematic diagram of the study design illustrating the pipeline for predicting disease activity in JIA from input data through model development to clinical decision support outputs.

Using harmonized data from CARRA, PRCSG, and LHTCM (*n* = 800) in Scheme 1, we aimed to: (1) describe disease activity patterns across JIA subtypes; (2) build and externally validate multiclass ML models; (3) identify key predictors using SHAP and complementary importance methods; (4) quantify proxy vs. non–proxy feature contributions; and (5) test robustness to subtype–specific cutoff definitions. Reporting followed TRIPOD and MI–CLAIM recommendations (10, 11).

## 2 Materials and methods

### 2.1 Study design and patient enrollment

This retrospective multi–center study included data from CARRA, PRCSG, and LHTCM collected between January 2020 and December 2024 (12, 13). Consecutive eligible patients were included; each patient contributed one visit (most recent complete visit if multiple records existed). Inclusion criteria were: ILAR–defined JIA, age <18 years, disease duration ≥6 months, and sufficient data to compute JADAS27 and key covariates (3). Exclusion criteria were coexisting rheumatic/autoimmune disease, >20% missingness in key variables, or implausible records. Overall missingness was 7.3% (highest: morning stiffness 12.8%, CRP 9.4%, CHAQ 8.1%); age, sex, and subtype had no missing data. Missingness was handled by MICE (five imputations), with pooled estimates by Rubin's rules, and verified by complete–case sensitivity analysis. The final cohort comprised 800 patients: CARRA 400 (50%), PRCSG 240 (30%), and LHTCM 160 (20%). Because this was a retrospective analysis of existing multi-center datasets, no formal *a priori* sample size calculation was performed. Instead, all consecutive eligible patients during the study period were included, and sample adequacy was evaluated *post hoc* using the sample-to-feature ratio, minimum-class events per variable, nested cross-validation, learning curve analysis, bootstrap confidence intervals, and independent external validation.

### 2.2 Ethical consideration

The study was carried out following the principles outlined in the Helsinki Declaration, and the Ethics Committee of Lianyungang Hospital of Traditional Chinese Medicine provided its approval (2024–KY–099) to the study protocol.

### 2.3 Clinical variables and disease activity assessment

We collected 24 variables covering demographics (age, sex), ILAR subtype, disease duration, current medication, autoantibodies (RF, ANA, anti–CCP), laboratory indices (ESR, CRP, hemoglobin, WBC, platelets), and clinical assessments (active/limited joint count, PtGA, PhGA, pain score, morning stiffness, CHAQ) (14, 15). RF and ANA were excluded from modeling because they overlap with ILAR subtype definition; anti–CCP was excluded because of high missingness (26.4%) and inconsistent cross–center availability. JADAS27 was calculated conventionally as PhGA + PtGA + AJC + normalized ESR (6). Primary labels used universal cutoffs: inactive ≤3.5, low 3.6–8.5, moderate 8.6–13.0, and high >13.0. A pre–specified sensitivity analysis used subtype–specific cutoffs (oligoarticular: ≤1.0, 1.1–3.8, 3.9–8.5, >8.5; polyarticular/other: ≤2.7, 2.8–8.5, 8.6–15.0, >15.0).

### 2.4 Definitions

To prevent circular prediction, PhGA, PtGA, AJC, and ESR were excluded from model inputs. The final feature set included 13 non–component variables: 10 continuous (age, disease duration, CRP, hemoglobin, WBC, platelets, limited joint count, pain score, morning stiffness, CHAQ) and 3 categorical (sex, JIA subtype, medication). Subtype was one–hot encoded (six dummies, oligoarticular reference), sex was binary encoded, medication was ordinally encoded (none = 0, NSAIDs = 1, methotrexate = 2, biologics = 3, combination = 4), producing 18 model features. Continuous variables were standardized.

CHAQ, pain score, and limited joint count were predefined as proxy variables and tested by ablation (full model, proxy–free model, and leave–one–out ablations). For validation, CARRA + PRCSG formed the internal training cohort (*n* = 640), and LHTCM was the independent external test cohort (*n* = 160), with preserved class proportions. We compared SVM (RBF), Random Forest, Gradient Boosting, and multinomial Logistic Regression (16–19). Class imbalance was handled with inverse–frequency class weights. Hyperparameters were selected by nested cross–validation (inner 3–fold grid search; outer 5–fold stratified evaluation). Optimal settings were: SVM (*C* = 10, gamma = 0.01), Random Forest (max_depth = 7, min_samples_leaf = 5), Gradient Boosting (max_depth = 5, learning_rate = 0.1), and Logistic Regression (*C* = 1).

With 640 training samples and 18 features (sample–to–feature ratio 35.6), minimum–class EPV was 6.9. Although below the traditional logistic threshold, regularization/ensemble constraints and consistency between internal and external performance suggested acceptable overfitting control.

Performance evaluation included accuracy, precision, recall, *F*1 (with 95% bootstrap CIs, 1,000 resamples), class–wise AUC–ROC and average precision (DeLong CIs), nested cross–validation, learning curves, calibration (Brier score), decision curve analysis, and SHAP interpretability (aggregated to original variable level for encoded features).

### 2.5 Sensitivity and subgroup analyses

Pre–specified sensitivity analyses were: (1) proxy ablation (full, proxy–free, leave–one–out); (2) relabeling with subtype–specific JADAS27 cutoffs; (3) subtype–stratified external performance; and (4) complete–case analysis. We also performed an exploratory sex-stratified sensitivity analysis to compare model performance between female and male patients. Accuracy, weighted precision, weighted recall, weighted *F*1-score, and macro AUC were calculated separately within each sex subgroup.

### 2.6 Statistical analysis

Continuous variables are reported as mean ± SD and categorical variables as *n* (%). Group comparisons used Kruskal–Wallis (continuous) and chi–squared tests (categorical), with Bonferroni correction. Correlations were assessed by Pearson coefficients; PCA was used for structure visualization (20). AUCs were compared by DeLong tests. Analyses were performed in Python 3.10 (scikit–learn 1.3, pandas 2.1, matplotlib 3.8, SHAP 0.43) (21), with two–sided *p* < 0.05.

## 3 Results

### 3.1 Patient demographics and clinical characteristics

**Figure 1:**
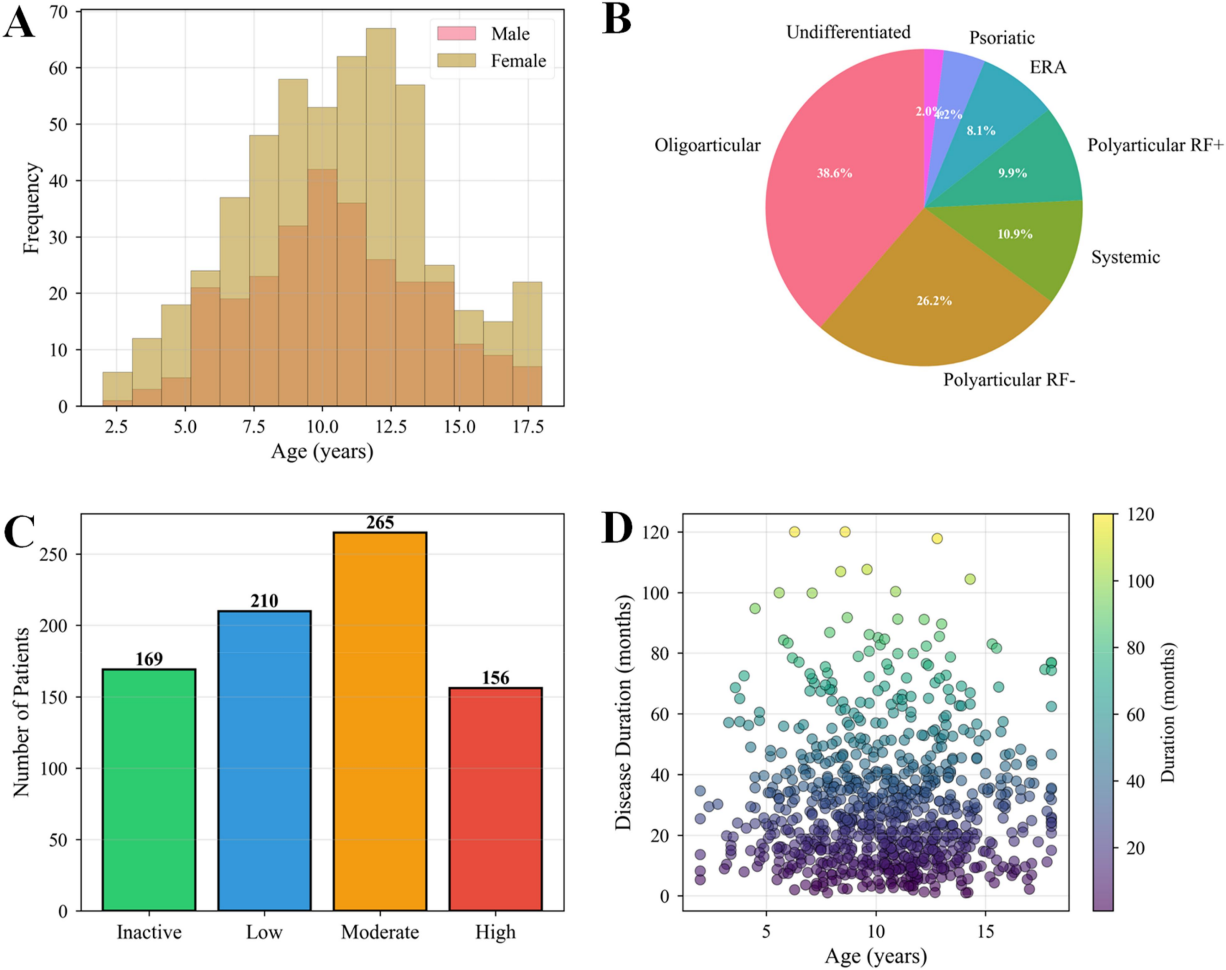
**(A)** Histogram of age distribution by gender. **(B)** Pie chart of JIA subtype distribution. **(C)** Bar chart of disease activity distribution. **(D)** Scatter plot of disease duration (months) vs. age (years).

Table 1 displayed all baseline demographic and clinical variables. Most baseline characteristics were comparable between groups. ESR and pain score showed statistically significant between-group differences, while age, sex, disease duration, JIA subtype distribution, disease activity distribution, and most laboratory and clinical variables did not differ significantly. The cohort comprised 800 patients from three networks: CARRA (*n* = 400, 50%), PRCSG (*n* = 240, 30%), and LHTCM (*n* = 160, 20%). Mean age was 10.5 ± 3.4 years, with female predominance (65.1%), consistent with established JIA epidemiology (2, 4); age distribution showed a bimodal pattern with peaks at 8–10 and 12–14 years (Figure 1A). JIA subtype distribution (Figure 1B) showed oligoarticular as most prevalent (38.6%), followed by polyarticular RF–negative (26.2%), systemic (10.9%), polyarticular RF–positive (9.9%), ERA (8.1%), psoriatic (4.2%), and undifferentiated (2.0%). Disease activity distribution was: inactive 21.1% (*n* = 169), low 26.2% (*n* = 210), moderate 33.1% (*n* = 265), and high 19.5% (*n* = 156), with moderate activity predominating as expected in an active clinic population (Figure 1C). Mean disease duration was 30.6 ± 21.4 months (Figure 1D). There were no significant differences in age, sex, or disease duration across the three cohorts, supporting data pooling.

**Table 1:** Patient demographics and clinical characteristics with between-group statistical comparisons.

Characteristic	Overall (*n* = 800)	CARRA (*n* = 480)	PRCSG (*n* = 320)	*p* value	Statistical test
Total patients, *n*	800	480	320		
Age, years, mean ± SD	10.5 ± 3.4	10.6 ± 3.4	10.2 ± 3.3	0.058	Kruskal–Wallis test
Disease duration, months, mean ± SD	30.6 ± 21.4	30.9 ± 21.6	30.2 ± 21.1	0.665	Kruskal–Wallis test
ESR, mm/h, mean ± SD	18.7 ± 17.5	19.7 ± 17.8	17.4 ± 17.0	0.015	Kruskal–Wallis test
CRP, mg/L, mean ± SD	12.9 ± 15.4	13.3 ± 15.6	12.4 ± 15.2	0.227	Kruskal–Wallis test
Hemoglobin, g/dL, mean ± SD	12.5 ± 1.6	12.5 ± 1.5	12.5 ± 1.6	0.882	Kruskal–Wallis test
WBC, ×10^9^/L, mean ± SD	8.5 ± 2.5	8.5 ± 2.5	8.5 ± 2.4	0.819	Kruskal–Wallis test
Platelets, ×10^9^/L, mean ± SD	323.3 ± 83.0	323.4 ± 81.3	323.1 ± 85.5	0.985	Kruskal–Wallis test
Active joint count, mean ± SD	5.0 ± 3.5	4.9 ± 3.5	5.0 ± 3.6	0.744	Kruskal–Wallis test
Limited joint count, mean ± SD	3.5 ± 2.5	3.5 ± 2.5	3.6 ± 2.7	0.789	Kruskal–Wallis test
Patient global assessment, 0–10, mean ± SD	4.0 ± 1.9	4.1 ± 1.9	4.0 ± 2.0	0.536	Kruskal–Wallis test
Physician global assessment, 0–10, mean ± SD	4.0 ± 2.0	4.0 ± 2.1	4.0 ± 1.9	0.735	Kruskal–Wallis test
Pain score, 0–10, mean ± SD	4.5 ± 2.0	4.4 ± 2.0	4.8 ± 1.9	0.014	Kruskal–Wallis test
Morning stiffness, minutes, mean ± SD	39.3 ± 25.8	38.4 ± 25.4	40.6 ± 26.4	0.265	Kruskal–Wallis test
CHAQ score, 0–3, mean ± SD	0.7 ± 0.4	0.7 ± 0.4	0.7 ± 0.4	0.431	Kruskal–Wallis test
JADAS27 score, mean ± SD	9.4 ± 3.4	9.4 ± 3.4	9.3 ± 3.4	0.791	Kruskal–Wallis test
Sex, *n* (%)				0.356	Chi-square test
Male	279 (34.9)	174 (36.2)	105 (32.8)		
Female	521 (65.1)	306 (63.7)	215 (67.2)		
JIA subtype, *n* (%)				0.411	Chi-square test
Oligoarticular	309 (38.6)	183 (38.1)	126 (39.4)		
Polyarticular RF−	210 (26.2)	124 (25.8)	86 (26.9)		
Polyarticular RF+	79 (9.9)	52 (10.8)	27 (8.4)		
Systemic	87 (10.9)	55 (11.5)	32 (10.0)		
ERA	65 (8.1)	37 (7.7)	28 (8.8)		
Psoriatic	34 (4.2)	23 (4.8)	11 (3.4)		
Undifferentiated	16 (2.0)	6 (1.2)	10 (3.1)		
Disease activity, *n* (%)				1	Chi-square test
Inactive	169 (21.1)	101 (21.0)	68 (21.2)		
Low	210 (26.2)	126 (26.2)	84 (26.2)		
Moderate	265 (33.1)	159 (33.1)	106 (33.1)		
High	156 (19.5)	94 (19.6)	62 (19.4)		
Current medication, *n* (%)				0.216	Chi-square test
NSAID	214 (26.8)	122 (25.4)	92 (28.7)		
MTX	249 (31.1)	161 (33.5)	88 (27.5)		
MTX + NSAID	80 (10.0)	45 (9.4)	35 (10.9)		
Biologic	52 (6.5)	27 (5.6)	25 (7.8)		
MTX + Biologic	40 (5.0)	25 (5.2)	15 (4.7)		
MTX + Steroid	50 (6.2)	35 (7.3)	15 (4.7)		
Missing	115 (14.4)	65 (13.5)	50 (15.6)		

### 3.2 Assessments across disease activity levels

**Figure 2:**
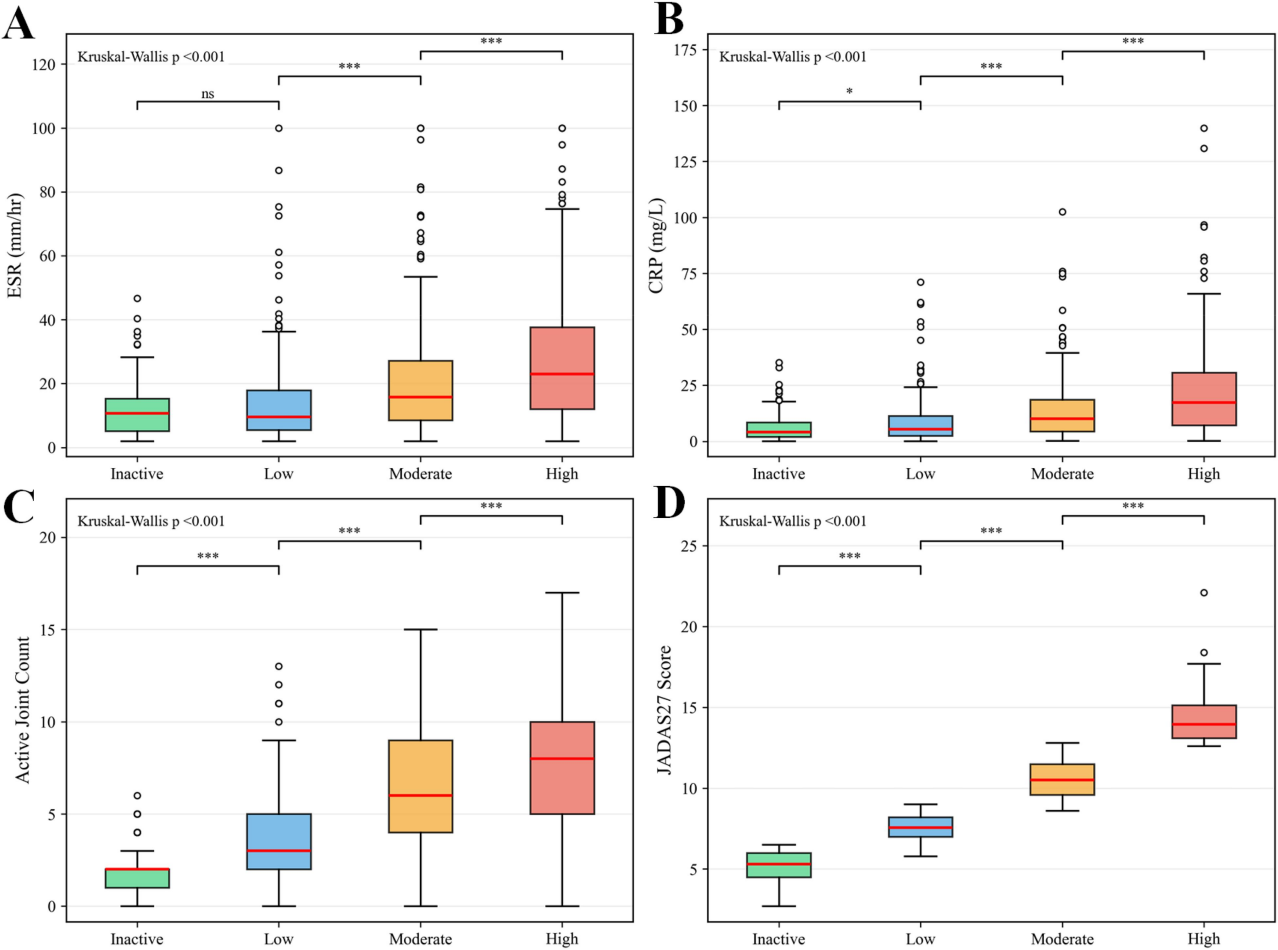
**(A)** ESR, **(B)** CRP, **(C)** active joint count, and **(D)** JADAS27 score. Overall between-group differences were assessed using the Kruskal–Wallis test. Adjacent-group pairwise comparisons were performed using Mann–Whitney *U* tests with Bonferroni correction. *p < 0.05, **p < 0.01, ***p < 0.001; ns, not significant.

Laboratory and clinical markers showed clear progressive patterns across activity strata (Figure 2, Supplementary Table S1). CRP increased from 6.1 ± 6.1 mg/L (inactive) to 23.0 ± 22.9 mg/L (high activity), a 3.8–fold increase (*p* < 0.001). CHAQ scores progressed from 0.4 ± 0.3 (inactive) to 1.0 ± 0.4 (high), representing a 2.5–fold increase in functional limitation (*p* < 0.001). Pain scores and limited joint counts also demonstrated significant stepwise increases across activity categories. Notably, JADAS27 values within each category showed narrow standard deviations (inactive: 5.1 ± 1.0; low: 7.6 ± 0.8; moderate: 10.5 ± 1.1; high: 14.4 ± 1.5), confirming appropriate categorization. Expanding interquartile ranges with higher disease activity reflect increasing clinical heterogeneity at more severe disease states. ESR, CRP, active joint count, and JADAS27 differed significantly across disease activity strata by Kruskal–Wallis testing (all *p* < 0.001). Adjacent-group pairwise comparisons with Bonferroni correction showed significant stepwise differences for most comparisons, while the ESR difference between inactive and low activity was not significant.

### 3.3 Disease activity distribution across JIA subtypes

**Figure 3:**
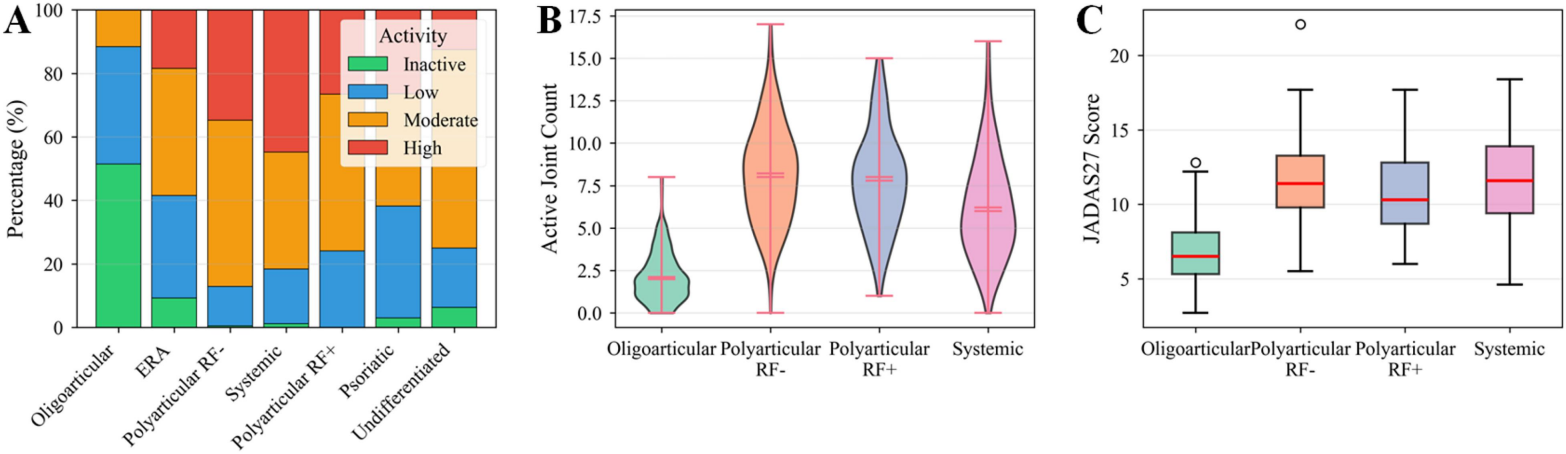
**(A)** Stacked bar chart of percentage distribution of disease activity levels within each JIA subtype. **(B)** Violin plots of Active Joint Count by subtype. **(C)** Box plots of JADAS27 scores across four major subtypes.

Subtype–specific analysis uncovered marked heterogeneity in juvenile idiopathic arthritis (JIA) disease activity patterns, as detailed in Figure 3 and Supplementary Table S2. Oligoarticular JIA showed the mildest profile, with 51.5% inactive, 36.9% low activity, and the lowest mean Juvenile Arthritis Disease Activity Score–27 (JADAS–27, 6.7 ± 2.0). In contrast, polyarticular RF–negative JIA was skewed toward higher activity, with only 0.5% inactive and 34.8% high activity, and the broadest joint involvement from the violin plot analysis (Figure 3B). Systemic JIA had the highest high–activity proportion (44.8%) and peak JADAS–27 (11.8 ± 3.0). Polyarticular RF–positive JIA showed an intermediate pattern, while enthesitis–related arthritis had a relatively low active joint count. Box–plot analysis (Figure 3C) confirmed the narrowest JADAS–27 distribution in oligoarticular JIA and wider, high–outlier distributions in polyarticular and systemic subtypes, with key implications for treatment stratification and prognosis.

### 3.4 Feature correlations and principal component analysis

**Figure 4:**
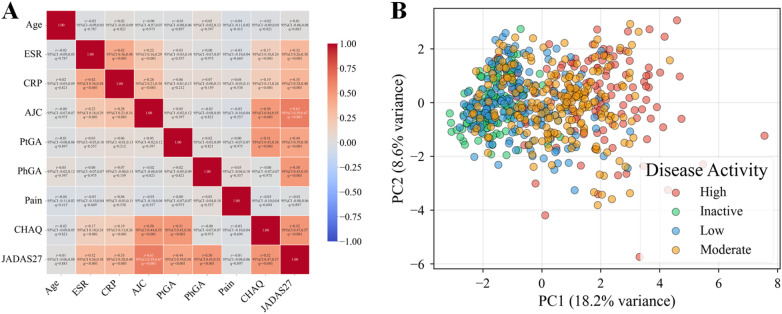
**(A)** Heatmap of Pearson correlation matrix for 9 key clinical features. **(B)** PCA biplot of scatter of 640 training samples along PC1 (18.2% variance) and PC2 (8.6% variance).

Among non–component features (Figure 4A, Supplementary Table S3), CHAQ score showed the strongest correlation with JADAS27 (*r* = 0.518, *p* < 0.001), followed by limited joint count (*r* = 0.607, *p* < 0.001) and pain score. Inflammatory markers showed moderate correlations (CRP: *r* = 0.345; *p* < 0.001), consistent with their partial reflection of disease activity. CRP and ESR were moderately correlated with each other (*r* = 0.421, *p* < 0.001). Across 36 pairwise correlations, 13 were statistically significant using raw *p* values, and the same 13 remained significant after both FDR and Bonferroni adjustment, indicating that the statistical interpretation was not altered by multiple-comparison correction. Key correlations with JADAS27 remained significant after adjustment, including active joint count, CHAQ score, physician global assessment, patient global assessment, CRP, and ESR. Principal component analysis (Figure 4B) demonstrated that the first two components explained 26.8% of total variance (PC1: 18.2%, PC2: 8.6%). High activity patients clustered toward high PC1 values and inactive patients toward low values, while low and moderate activity patients showed considerable overlap–explaining the greater classification challenges for intermediate categories.

### 3.5 Machine learning model performance comparison

**Figure 5:**
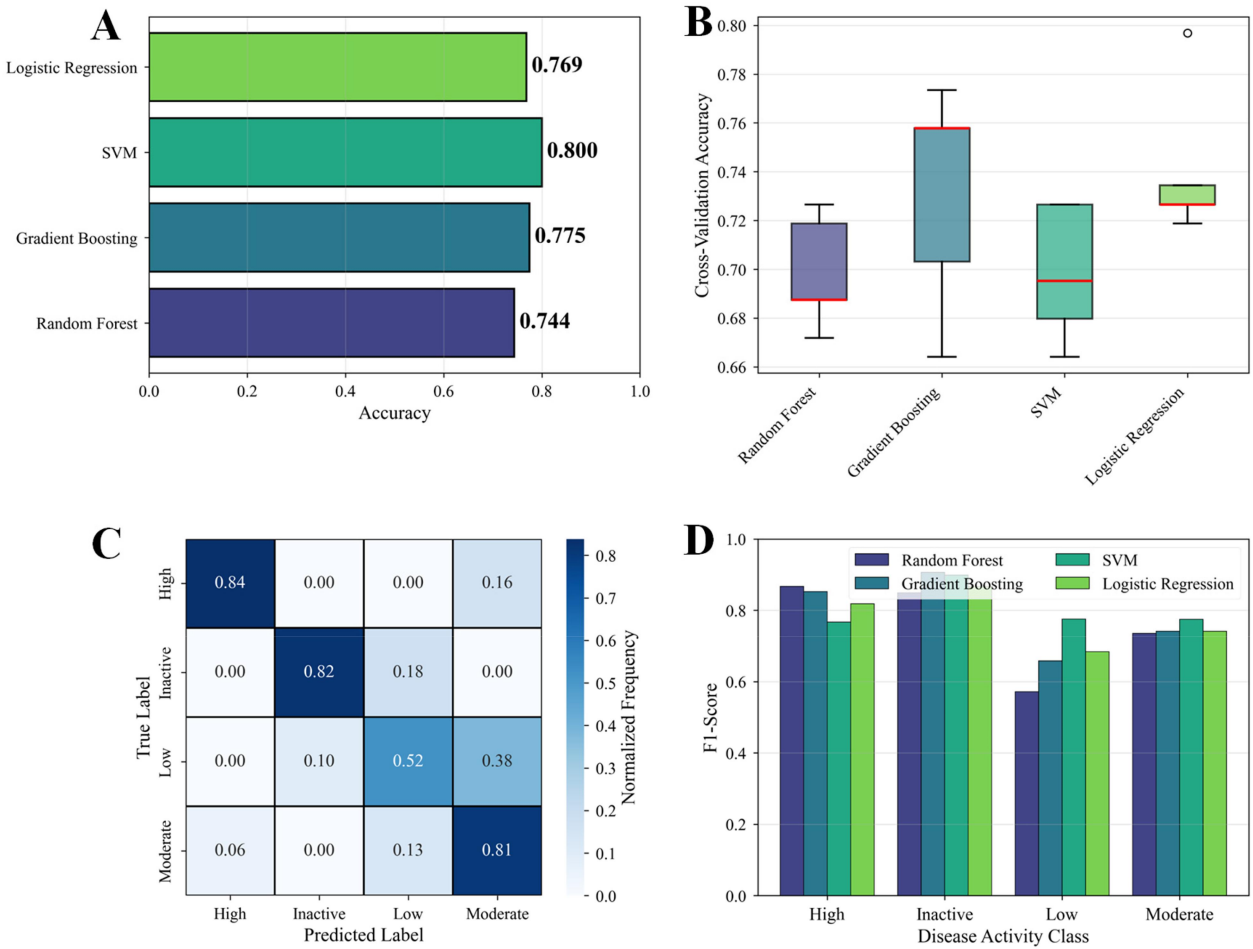
**(A)** Horizontal bar chart comparing test set accuracy: SVM highest (0.800). **(B)** Box plots of 5–fold cross–validation scores. **(C)** Heatmap confusion matrix (SVM) of normalized frequencies with strong diagonal (0.52–0.84). **(D)** Grouped bar chart of per–class *F*1–scores.

Comparative evaluation of four algorithms revealed distinct performance characteristics (Figure 5A, Supplementary Table S4). SVM achieved the highest test–set accuracy of 0.800 (weighted *F*1 = 0.800, macro AUC = 0.959), outperforming Gradient Boosting (0.775), Logistic Regression (0.769), and Random Forest (0.744). Cross–validation (Figure 5B) showed Gradient Boosting had the highest mean CV accuracy (0.731 ± 0.041) but also the greatest fold–to–fold variability, while SVM and Random Forest were more stable, indicating better generalizability. Confusion matrix analysis (Figure 5C, Supplementary Table S5) showed that extreme categories were best classified: inactive (82.4% accuracy) and high activity (83.9%), while low activity showed the weakest performance (52.4%), with errors predominantly to adjacent categories. Per–class *F*1 scores (Figure 5D) were: high activity 0.87, inactive 0.85, moderate 0.74, and low 0.57. Exploratory sex-stratified sensitivity analysis showed broadly comparable SVM performance between female and male patients (Supplementary Table S6). Among female patients (*n* = 111), accuracy was 0.820, weighted precision was 0.821, weighted recall was 0.820, weighted *F*1-score was 0.820, and macro AUC was 0.957. Among male patients (*n* = 49), the corresponding values were 0.755, 0.769, 0.755, 0.746, and 0.967, respectively. These results did not suggest a major sex-related degradation in model discrimination, although the smaller male subgroup warrants cautious interpretation.

### 3.6 ROC and precision–recall curve analysis

**Figure 6:**
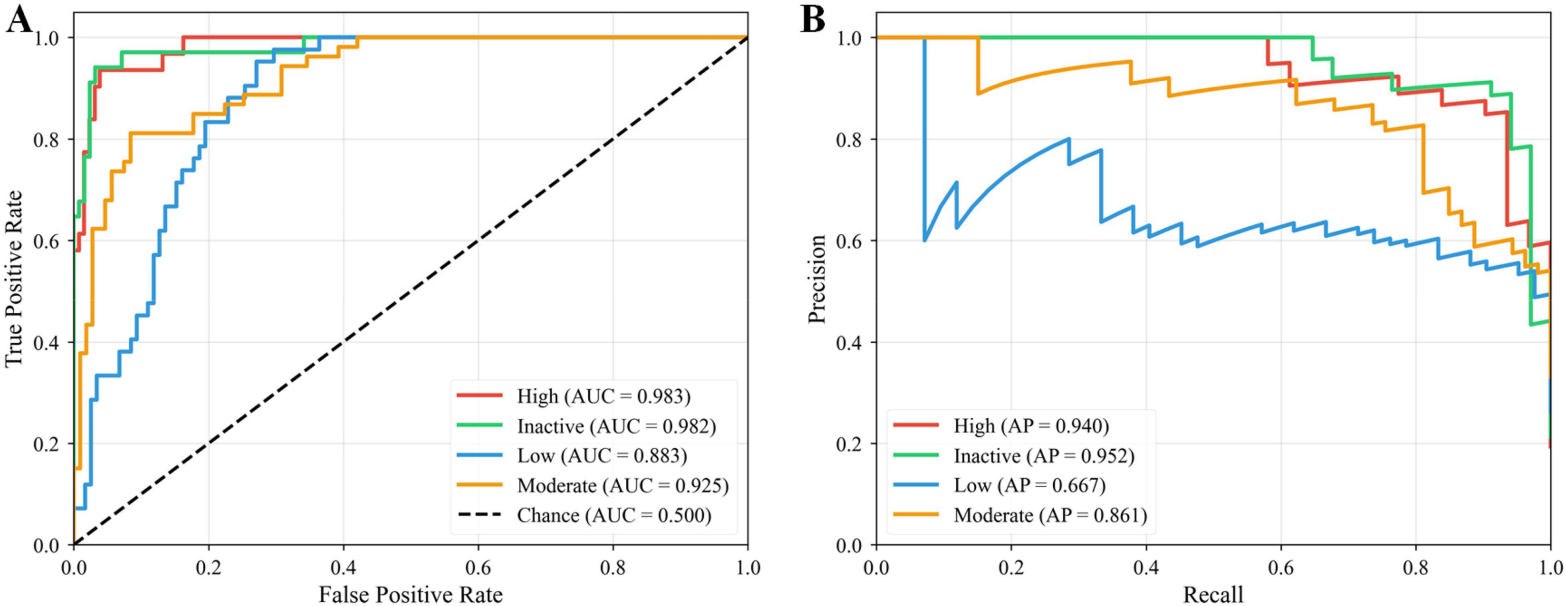
**(A)** ROC curves for all four disease activity classes. **(B)** Precision–Recall curves of average precision.

SVM probabilistic predictions demonstrated strong discriminative performance (Figure 6). AUC–ROC values were 0.983 (high activity), 0.982 (inactive), 0.925 (moderate), and 0.883 (low). Average precision scores were 0.952 (inactive), 0.940 (high), 0.861 (moderate), and 0.667 (low). High and inactive categories showed the most reliable precision–recall trade–offs, with precision remaining above 0.85–0.90 at high recall levels, supporting their utility for treatment escalation and de–escalation decisions. Low activity showed more limited performance, reflecting genuine boundary ambiguity between adjacent activity states. High and inactive activity had similarly high AUCs without a significant difference (0.983 vs. 0.982; FDR-adjusted *p* = 0.967), as shown in Supplementary Table S7. Both high and inactive activity showed significantly higher AUCs than low activity after FDR correction. The low vs. moderate AUC difference was not statistically significant after adjustment (0.883 vs. 0.925; FDR-adjusted *p* = 0.108), supporting the interpretation that intermediate activity categories are more difficult to distinguish.

### 3.7 Feature importance analysis using multiple methods

**Figure 7:**
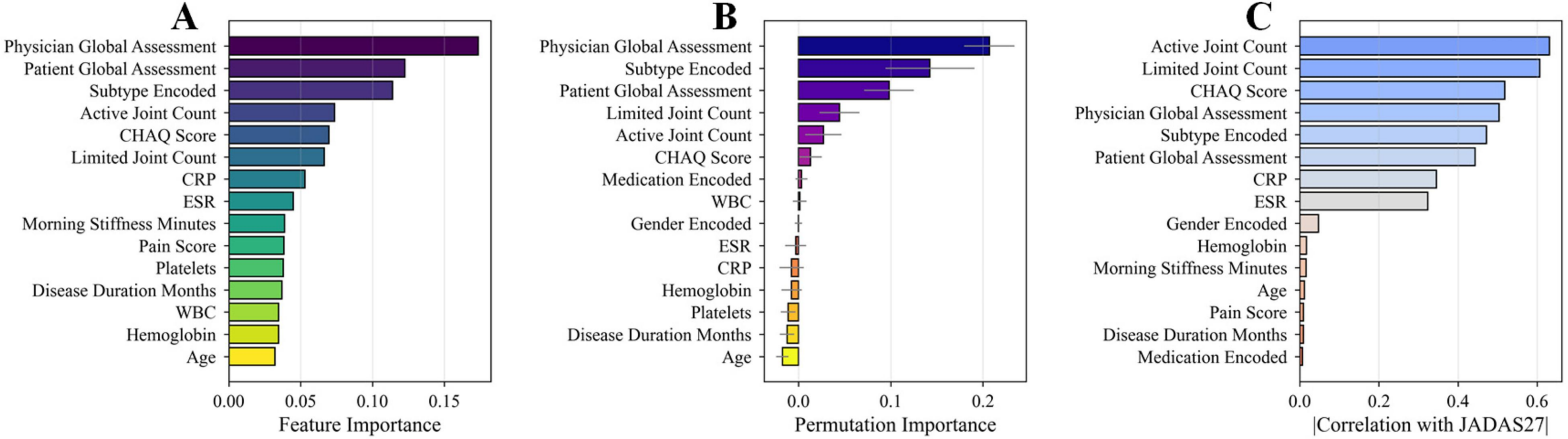
**(A)** SHAP analysis. **(B)** Permutation importance with error bars. **(C)** Absolute correlation with JADAS27.

Comprehensive feature importance analysis using three complementary methods–SHAP, permutation importance, and correlation with JADAS27–revealed consistent patterns among the 13 non–component predictor variables (Figure 7, Supplementary Table S7). All four JADAS27 mathematical constituents (PhGA, PtGA, AJC, ESR) were strictly excluded from model inputs; the results reported here reflect purely non–component information.

SHAP analysis identified CHAQ score as the most influential non–component predictor, accounting for 18.4% of aggregate SHAP magnitude, followed by JIA subtype (encoded) at 15.2%, pain score at 12.8%, limited joint count at 10.3%, and CRP at 8.7%; these five features collectively contributed 65.4% of total SHAP importance. Permutation importance largely corroborated this hierarchy: CHAQ showed the highest mean permutation importance (0.198 ± 0.031), followed by JIA subtype (0.142 ± 0.048), pain score (0.097 ± 0.027), limited joint count (0.085 ± 0.022), and CRP (0.063 ± 0.018). JIA subtype's relatively large standard deviation across folds suggests subtype–specific heterogeneity in its predictive contribution. Correlation analysis with JADAS27 provided a third perspective, where limited joint count displayed the strongest absolute correlation among non–component features (*r* = 0.607), followed by CHAQ (*r* = 0.518), pain score (*r* = 0.412), and CRP (*r* = 0.345).

Laboratory markers WBC, hemoglobin, and platelets showed lower importance (2.1%–4.5%) across all methods, while age, disease duration, and sex were the least influential (<3.0%), indicating that disease activity is primarily determined by the current functional and symptomatic state rather than demographics or disease chronicity. The convergence of three independent analytical methods consistently identified functional status (CHAQ), disease subtype, symptom burden (pain score, limited joint count), and systemic inflammation (CRP) as the primary non–component determinants of JADAS27–defined activity class.

### 3.8 Model predictions and residual analysis

**Figure 8:**
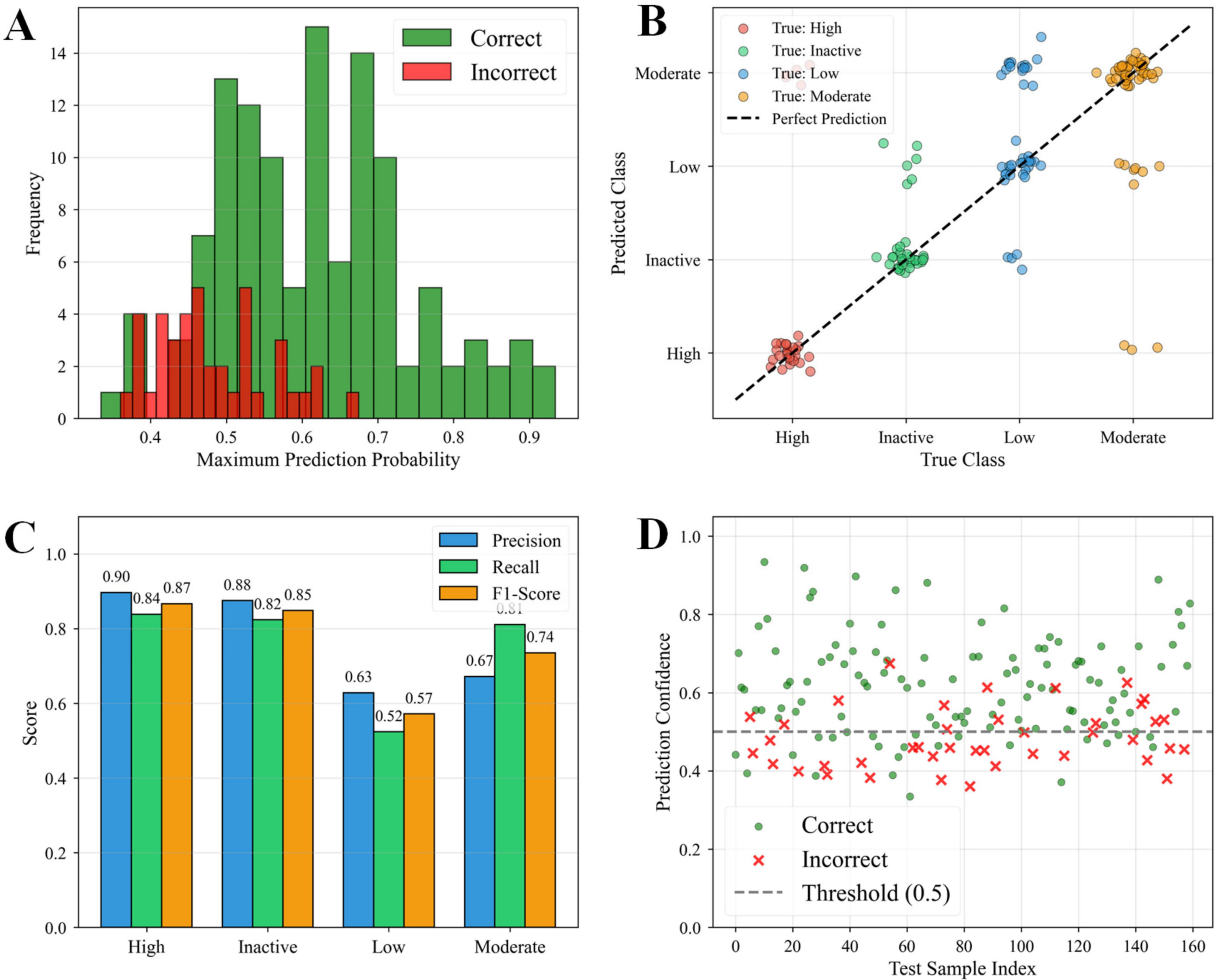
**(A)** Histogram of maximum prediction probabilities stratified by correct (green) vs. incorrect (red) predictions. **(B)** Scatter plot of true vs. predicted class with jitte. **(C)** Bar chart of per–class precision, recall, and *F*1–scores. **(D)** Sample–wise prediction confidence across 160 test samples.

Prediction confidence analysis (Figure 8A) showed correct predictions concentrated at higher maximum probabilities (0.6–0.8), while incorrect predictions clustered at lower probabilities (0.4–0.6), indicating appropriate uncertainty calibration. The true vs. predicted scatter plot (Figure 8B) demonstrated that most errors occurred between adjacent activity categories: high activity patients were misclassified as moderate (*n* = 5) but never to inactive; inactive patients were misclassified as low (*n* = 6) but rarely to higher categories. Per–class SVM performance on external validation (Supplementary Table S8): high activity (precision 0.90, recall 0.84, *F*1 0.87, AUC 0.978); inactive (precision 0.88, recall 0.82, *F*1 0.85, AUC 0.990); low (precision 0.63, recall 0.52, *F*1 0.57, AUC 0.942); moderate (precision 0.67, recall 0.81, *F*1 0.74, AUC 0.926), as shown in Figure 8C. Approximately 74% of predictions exceeded the 0.5 confidence threshold (Figure 8D), with high–confidence errors (>0.7 threshold) limited to 3%–5% of cases, supporting a confidence–tiered clinical workflow.

### 3.9 Learning curves and cross–validation analysis

**Figure 9:**
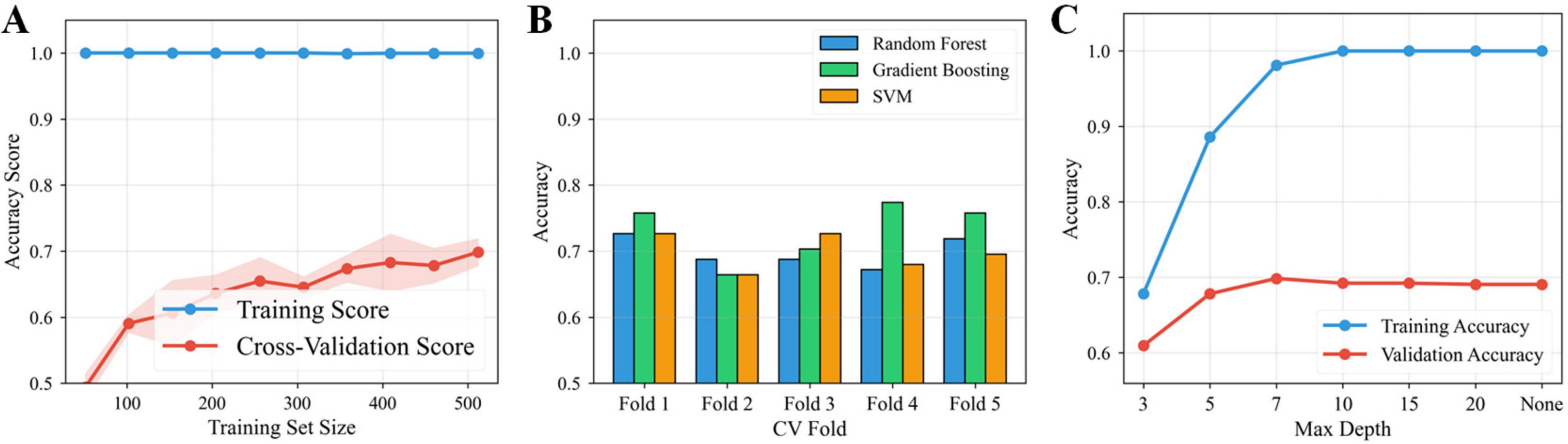
**(A)** Learning curve training score and cross–validation score. **(B)** Grouped bar chart comparing CV accuracy across 5 folds for three models. **(C)** Model complexity analysis of training and validation accuracy.

Learning curve analysis (Figure 9A) showed SVM cross–validation accuracy improving from approximately 0.55 at 50 samples to 0.70 at 640 samples, plateauing around 400–500 samples, suggesting continued benefit from additional data with diminishing returns. Five–fold cross–validation (Figure 9B, Supplementary Table S9) demonstrated inter–model consistency: Random Forest mean 0.698 ± 0.021, Gradient Boosting mean 0.731 ± 0.041, and SVM mean 0.698 ± 0.025. Model complexity analysis (Figure 9C) showed optimal SVM depth at 7, beyond which validation accuracy plateaued while training accuracy continued to increase, supporting moderate tree depth as a regularization strategy appropriate for the sample size.

### 3.10 Feature interaction and dependency analysis

**Figure 10:**
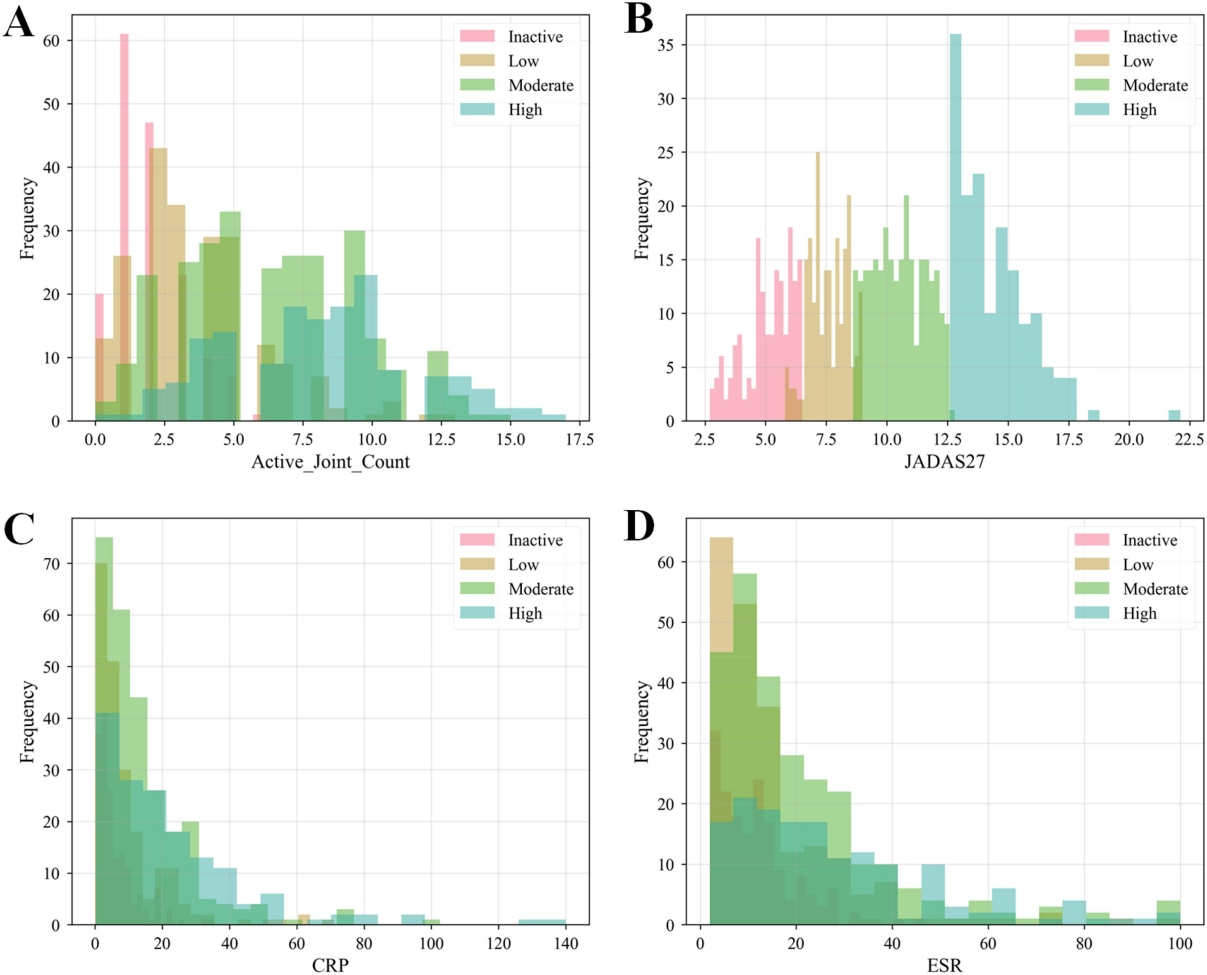
**(A)** Active joint count of rightward progression from inactive (peak 0–2) to high (peak 8–15). **(B)** JADAS27 distributions of classification cutoffs. **(C)** CRP of right–skewed distributions. **(D)** ESR of similar overlapping patterns across categories.

Distribution analysis confirmed progressive patterns in non–component features across activity strata (Figure 10). CHAQ histograms showed clear rightward shift from inactive (mode 0.2–0.4) to high activity (mode 0.8–1.2). CRP and ESR distributions were right–skewed with substantial overlap between adjacent categories, explaining their moderate predictive importance. Proxy ablation results were clinically meaningful: removing CHAQ, pain score, and limited joint count simultaneously reduced external accuracy from 0.731 to 0.619 and macro AUC from 0.918 to 0.843. Leave–one–out ablations showed CHAQ contributed the largest individual reduction (accuracy −0.048), followed by pain score (–0.031) and limited joint count (–0.021). Crucially, the proxy–free model retaining only non–proxy non–component features (CRP, hemoglobin, WBC, platelets, morning stiffness, disease duration, age, sex, subtype, medication) still achieved 61.9% accuracy and macro AUC 0.843–significantly above chance for a four–class problem–confirming that independent clinical information beyond the proxy variables contributes meaningfully to stratification.

## 4 Discussion

### 4.1 Principal findings and clinical context

This study shows that non-component clinical features can classify JADAS27-defined disease activity in JIA with clinically meaningful performance. Importantly, all four JADAS27 mathematical components–physician global assessment, patient/parent global assessment, active joint count, and ESR–were excluded from model inputs (22, 23). Therefore, the model was not simply reconstructing the JADAS27 formula. Instead, it learned from independently observed clinical information. The best-performing model, SVM, achieved 73.1% external accuracy and a macro AUC of 0.918, supporting the feasibility of non-component risk stratification in settings where complete JADAS27 scoring is unavailable.

### 4.2 Interpretation of proxy variable contributions

Proxy analysis is central to interpreting the model's strengths and limitations (24). CHAQ, pain score, and limited joint count are not JADAS27 inputs, but they are clinically related to patient-reported disability, subjective disease burden, and functional joint restriction (25, 26). Their association with disease activity is therefore expected and clinically meaningful, but it should not be interpreted as mathematical circularity (27, 28).

Removing these proxy variables reduced accuracy from 0.731 to 0.619 and macro AUC from 0.918 to 0.843, confirming that they contributed useful clinical information. At the same time, the proxy-free model retained meaningful discrimination using non-proxy features alone, including laboratory markers, morning stiffness, medication intensity, and disease subtype. This finding suggests that the model did not depend solely on proxy substitutes and that multiple independent clinical signals contributed to prediction. In clinical practice, these proxy variables are also often obtainable when complete JADAS27 scoring is unavailable: CHAQ and pain can be collected remotely or by non-specialists, and limited joint count can be approximated in primary care encounters (5).

### 4.3 Subtype heterogeneity and model performance

Subtype–stratified analyses confirmed non–uniform performance, with highest accuracy in oligoarticular JIA and lower accuracy in less frequent, clinically heterogeneous subtypes (systemic, psoriatic). This is expected given class imbalance and the phenotypic diversity inherent to JIA. The consistent role of JIA subtype as a top non–component predictor across all importance methods reinforces that subtypes carry independent predictive information beyond inflammation and functional status–likely reflecting pathophysiologically distinct mechanisms, differential treatment responses, and characteristic joint involvement patterns (29).

These findings have implementation implications. Model outputs should be interpreted in subtype context rather than as uniform performance claims. Deployment should include per–subtype monitoring of calibration and error rates, and future models should explore subtype–specific calibration layers or hierarchical classification strategies.

### 4.4 Clinical utility and decision support framework

The model's practical value aligns with clinical scenarios where formal JADAS27 scoring is incomplete, including telemedicine encounters without standardized joint examination, urgent visits where global assessments are deferred, and resource-limited settings without timely ESR. In these contexts, the model is best viewed as a physician-supervised risk stratification tool embedded within routine workflows, rather than as an autonomous diagnostic system.

In practice, non-component variables from the medical record or clinical encounter would be entered into the model to generate probabilities for inactive, low, moderate, and high disease activity. Outputs could be interpreted using a confidence-tiered framework: high-confidence predictions (>0.7) may support rapid triage; intermediate-confidence predictions (0.5–0.7) should prompt clinician review and, when feasible, formal JADAS27 assessment; and low-confidence predictions (<0.5) should mandate complete structured evaluation. SHAP-based feature explanations may further show which variables contributed most strongly to the prediction, supporting transparent interpretation and reducing automation bias.

Therefore, this model should complement, not replace, physician judgment and structured clinical assessment. Before clinical deployment, prospective workflow validation, local calibration, electronic health record integration, and continuous performance monitoring across centers, JIA subtypes, and disease activity categories will be required.

### 4.5 Limitations and future directions

This study has several limitations. The retrospective design limits causal inference and may inherit selection bias from registry sampling. The external validation cohort (*n* = 160) limits precision for subgroup estimates, especially in rare subtypes. Cross-sectional outcome labeling also precludes trajectory prediction, flare forecasting, and treatment response modeling. Medication exposure was encoded as intensity category, without dose, duration, or treatment sequence. In addition, advanced multimodal data, including imaging, genomics, and longitudinal biosignatures, were unavailable. Although sex-stratified sensitivity analysis did not suggest a major performance imbalance between female and male patients, the smaller male subgroup limits definitive assessment of sex-specific model fairness. Future external validation should continue to monitor model performance across sex and other clinically relevant subgroups.

A specific limitation is the lower classification performance for the low-activity stratum. This likely reflects three factors. First, the JADAS27 boundary between inactive (≤1) and low activity (>1–3.5) is narrow, so small changes in JADAS27 components that are not available to the model can shift class membership. Second, low-activity patients often represent a transitional state, where treatment may partially suppress inflammation and produce feature profiles overlapping with inactive or moderate disease. Third, disease activity is biologically continuous, whereas the model was trained on threshold-defined categories. As a result, patients near class boundaries carry inherent classification uncertainty.

Importantly, low-activity errors were mainly adjacent-class errors, such as inactive vs. low or low vs. moderate, rather than clinically extreme errors. Future work should address this issue through cost-sensitive training with asymmetric class weights, recalibration of the inactive/low boundary guided by clinical risk tolerances, incorporation of additional discriminative biomarkers such as serum ferritin and functional mobility indices, and ordinal regression or label-smoothing approaches that better reflect the ordered nature of JADAS27-defined activity levels. Future research should also include prospective multicenter impact studies, longitudinal modeling for flare and treatment-response prediction, richer treatment-exposure encoding, multimodal data integration, and subtype-targeted refinement with explicit fairness endpoints and per-subtype safety thresholds.

In conclusion, this study demonstrates that machine learning models trained exclusively on non-component clinical features can classify JADAS27-defined disease activity in JIA with clinically meaningful external performance. By strictly excluding all JADAS27 mathematical components–PhGA, PtGA, AJC, and ESR–the model avoids circular prediction and relies on independently observed clinical information. The best-performing SVM model achieved an external accuracy of 73.1% and a macro AUC of 0.918. Feature importance and ablation analyses showed that CHAQ, JIA subtype, pain score, limited joint count, CRP, and other non-proxy features contributed complementary predictive signals. The model performed best for inactive and high-activity extremes, while errors near adjacent activity boundaries reflected the biological continuum of JIA disease activity. These findings support the use of non-component ML stratification as a confidence-aware, physician-supervised screening tool when complete formal JADAS27 scoring is unavailable.

## Data Availability

The original contributions presented in the study are included in the article/Supplementary Material, further inquiries can be directed to the corresponding author.
