# Supplementary material for: Non–component clinical feature–based machine learning for disease activity risk stratification in juvenile idiopathic arthritis: a multi–center retrospective validation study
Source: Front Pediatr. 2026 Jul 15;14:1820800. doi: 10.3389/fped.2026.1820800 (PMC13415689; doi:10.3389/fped.2026.1820800)
Supplement: Supplementary file 1 [file Table1.docx]

**Supplementary materials**

**Non–Component Clinical Feature–Based Machine Learning for Disease Activity Risk Stratification in Juvenile Idiopathic Arthritis: A Multi–Center Retrospective Validation Study**

Peipei Dong^1^, Fei Song^2^, Bin Wang^3^, Song Gao^3^, Hongyang Dong^3^, Xiaohong Jiang^3^, Yan Cong^3^, Chuansheng Wu^3,*^

^1^ Pediatric Department, Lianyungang Hospital of Traditional Chinese Medicine, Lianyungang, Jiangsu, 222000, China

^2^ Traditional Chinese Medicine Department, The First People’s Hospital of Lianyungang, Lianyungang, Jiangsu, 222000, China

^3^ Department of Rheumatology, Lianyungang Hospital of Traditional Chinese Medicine, Lianyungang, Jiangsu, 222000, China

^*^ Corresponding author: Chuansheng Wu (Wucs_tcmedu@163.com)

Table S1. Laboratory markers and clinical assessments by disease activity.

| **Clinical Feature** | **Inactive** | **Low** | **Moderate** | **High** |
| --- | --- | --- | --- | --- |
| ESR (mm/hr) | 11.8 ± 8.4 (10.7 [5.2–15.3]) | 14.8 ± 14.8 (9.6 [5.5–17.9]) | 20.7 ± 18.3 (15.8 [8.5–27.2]) | 28.3 ± 21.7 (23.0 [12.1–37.6]) |
| CRP (mg/L) | 6.1 ± 6.1 (4.1 [2.1–8.4]) | 9.5 ± 11.1 (5.5 [2.6–11.4]) | 14.1 ± 13.8 (10.1 [4.5–18.6]) | 23.0 ± 22.9 (17.4 [7.1–30.7]) |
| Active Joint Count | 1.8 ± 1.3 (2.0 [1.0–2.0]) | 3.6 ± 2.5 (3.0 [2.0–5.0]) | 6.2 ± 3.1 (6.0 [4.0–9.0]) | 8.1 ± 3.4 (8.0 [5.0–10.0]) |
| JADAS27 Score | 5.1 ± 1.0 (5.3 [4.5–6.0]) | 7.6 ± 0.8 (7.5 [7.0–8.2]) | 10.5 ± 1.1 (10.5 [9.6–11.5]) | 14.4 ± 1.5 (13.9 [13.1–15.1]) |
| Patient Global Assessment | 3.2 ± 1.6 (3.1 [1.9–4.3]) | 3.5 ± 1.9 (3.1 [2.1–4.7]) | 4.2 ± 1.8 (4.1 [2.9–5.3]) | 5.3 ± 1.8 (5.4 [4.1–6.6]) |
| Physician Global Assessment | 2.8 ± 1.5 (2.6 [1.5–3.8]) | 3.7 ± 1.9 (3.6 [2.2–5.2]) | 4.0 ± 1.9 (3.8 [2.6–5.3]) | 5.5 ± 1.8 (5.7 [4.3–7.0]) |
| CHAQ Score | 0.4 ± 0.3 (0.4 [0.2–0.6]) | 0.6 ± 0.3 (0.5 [0.3–0.8]) | 0.7 ± 0.3 (0.7 [0.5–1.0]) | 1.0 ± 0.4 (1.0 [0.8–1.3]) |

Table S2. Disease activity distribution by JIA subtype.

| **JIA Subtype** | **Total n (%)** | **Inactive n (%)** | **Low n (%)** | **Moderate n (%)** | **High n (%)** | **JADAS27 (mean ± SD)** | **Active Joints (mean ± SD)** |
| --- | --- | --- | --- | --- | --- | --- | --- |
| Oligoarticular | 309 (38.6) | 159 (51.5) | 114 (36.9) | 36 (11.7) | 0 (0.0) | 6.7 ± 2.0 | 2.1 ± 1.5 |
| Polyarticular RF– | 210 (26.2) | 1 (0.5) | 26 (12.4) | 110 (52.4) | 73 (34.8) | 11.5 ± 2.7 | 8.2 ± 2.9 |
| Polyarticular RF+ | 79 (9.9) | 0 (0.0) | 19 (24.1) | 39 (49.4) | 21 (26.6) | 10.7 ± 2.7 | 7.8 ± 2.9 |
| Systemic | 87 (10.9) | 1 (1.1) | 15 (17.2) | 32 (36.8) | 39 (44.8) | 11.8 ± 3.0 | 6.2 ± 3.0 |
| ERA | 65 (8.1) | 6 (9.2) | 21 (32.3) | 26 (40.0) | 12 (18.5) | 9.7 ± 3.1 | 3.4 ± 1.6 |
| Psoriatic | 34 (4.2) | 1 (2.9) | 12 (35.3) | 12 (35.3) | 9 (26.5) | 10.3 ± 3.4 | 4.4 ± 2.1 |
| Undifferentiated | 16 (2.0) | 1 (6.2) | 3 (18.8) | 10 (62.5) | 2 (12.5) | 10.0 ± 2.4 | 3.8 ± 1.9 |

Table S3. Feature correlation with disease activity markers.

| **Feature** | **Correlation with JADAS27** | **Correlation with ESR** | **Correlation with CRP** | **Correlation with Active Joint Count** |
| --- | --- | --- | --- | --- |
| Age | 0.012 | –0.021 | 0.016 | –0.004 |
| ESR | 0.323 |  | 0.421 | 0.223 |
| CRP | 0.345 | 0.421 |  | 0.279 |
| Active Joint Count | 0.631 | 0.223 | 0.279 |  |
| Patient Global Assessment | 0.443 | 0.035 | 0.06 | 0.047 |
| Physician Global Assessment | 0.504 | 0.001 | 0.066 | –0.015 |
| Pain Score | –0.009 | –0.028 | 0.038 | –0.034 |
| CHAQ Score | 0.518 | 0.17 | 0.194 | 0.497 |
| JADAS27 | 1 | 0.323 | 0.345 | 0.631 |

Table S4. Machine learning model performance comparison.

| Model | Accuracy | Precision (weighted) | Recall (weighted) | F1–Score (weighted) | AUC–ROC (macro) | CV Accuracy (mean ± SD) | Test Set Size |
| --- | --- | --- | --- | --- | --- | --- | --- |
| SVM | 0.8 | 0.802 | 0.8 | 0.8 | 0.959 | 0.698 ± 0.025 | 160 |
| Gradient Boosting | 0.775 | 0.784 | 0.775 | 0.776 | 0.938 | 0.731 ± 0.041 | 160 |
| Logistic Regression | 0.769 | 0.769 | 0.769 | 0.767 | 0.948 | 0.741 ± 0.029 | 160 |
| Random Forest | 0.744 | 0.747 | 0.744 | 0.742 | 0.943 | 0.698 ± 0.021 | 160 |

Table S5. Confusion matrix for disease activity classification.

| **True Class** | **Predicted High** | **Predicted Inactive** | **Predicted Low** | **Predicted Moderate** | **Total** | **Class Accuracy (%)** |
| --- | --- | --- | --- | --- | --- | --- |
| High | 26 | 0 | 0 | 5 | 31 | 83.9 |
| Inactive | 0 | 28 | 6 | 0 | 34 | 82.4 |
| Low | 0 | 4 | 22 | 16 | 42 | 52.4 |
| Moderate | 3 | 0 | 7 | 43 | 53 | 81.1 |
| Total Predicted | 29 | 32 | 35 | 64 | 160 | 74.4 |

Table S6. Test set predictive performance stratified by gender subgroups.

| **Sex** | **Test Set Size (n)** | **Accuracy** | **Precision (weighted)** | **Recall (weighted)** | **F1-Score (weighted)** | **AUC-ROC (macro)** |
| --- | --- | --- | --- | --- | --- | --- |
| Female | 111 | 0.82 | 0.821 | 0.82 | 0.82 | 0.957 |
| Male | 49 | 0.755 | 0.769 | 0.755 | 0.746 | 0.967 |

Table S7. Pairwise AUC comparisons across four activity grades with FDR correction.

| **Comparison** | **Class 1 AUC** | **Class 2 AUC** | **AUC difference** | **95% CI lower** | **95% CI upper** | **Raw p value** | **FDR-adjusted p value** | **Significant after FDR** |
| --- | --- | --- | --- | --- | --- | --- | --- | --- |
| High vs Inactive | 0.983 | 0.982 | 0.001 | -0.024 | 0.03 | 0.967 | 0.967 | No |
| High vs Low | 0.983 | 0.883 | 0.1 | 0.051 | 0.157 | <0.001 | <0.001 | Yes |
| High vs Moderate | 0.983 | 0.925 | 0.058 | 0.021 | 0.101 | 0.001 | 0.002 | Yes |
| Inactive vs Low | 0.982 | 0.883 | 0.099 | 0.058 | 0.15 | <0.001 | <0.001 | Yes |
| Inactive vs Moderate | 0.982 | 0.925 | 0.057 | 0.013 | 0.106 | 0.012 | 0.017 | Yes |
| Low vs Moderate | 0.883 | 0.925 | -0.042 | -0.094 | 0.005 | 0.09 | 0.108 | No |

Table S8. Per–class performance metrics (SVM – best overall model).

| **Disease Activity Class** | **Precision** | **Recall (Sensitivity)** | **F1–Score** | **AUC–ROC** | **Average Precision** | **Specificity** | **Support (n)** |
| --- | --- | --- | --- | --- | --- | --- | --- |
| High | 0.793 | 0.742 | 0.767 | 0.978 | 0.918 | 0.953 | 31 |
| Inactive | 0.886 | 0.912 | 0.899 | 0.99 | 0.959 | 0.968 | 34 |
| Low | 0.816 | 0.738 | 0.775 | 0.942 | 0.844 | 0.941 | 42 |
| Moderate | 0.741 | 0.811 | 0.775 | 0.926 | 0.834 | 0.86 | 53 |

Table S9. Cross–validation performance by fold.

| **CV Fold** | **Random Forest** | **Gradient Boosting** | **SVM** |
| --- | --- | --- | --- |
| Fold 1 | 0.727 | 0.758 | 0.727 |
| Fold 2 | 0.688 | 0.664 | 0.664 |
| Fold 3 | 0.688 | 0.703 | 0.727 |
| Fold 4 | 0.672 | 0.773 | 0.68 |
| Fold 5 | 0.719 | 0.758 | 0.695 |
| Mean ± SD | 0.698 ± 0.021 | 0.731 ± 0.041 | 0.698 ± 0.025 |
